# Genotype-Phenotype Correlations in Denys-Drash Syndrome in Children

**DOI:** 10.1016/j.ekir.2025.01.014

**Published:** 2025-01-16

**Authors:** Mathilde Glénisson, Mathilde Grapin, Thomas Blanc, Evgenia Preka, Julien Hogan, Manon Aurelle, Gwenaëlle Roussey, Antoine Mouche, Caroline Rousset-Rouviere, Robert Novo, Camille Faudeux, Marc Fila, Isabelle Vrillon, Sylvie Cloarec, Thomas Simon, Jérôme Harambat, Edouard Martinez Casado, Julien Rod, Morgane Carre Lecoindre, Laurence Heidet, Olivia Boyer, Nicolas Garcelon, Jessica Kachmar, Guillaume Dorval, Sabine Sarnacki

**Affiliations:** 1 Service de chirurgie viscérale, urologie et transplantation, Hôpital Necker-Enfants malades, GH Centre, Assistance Publique-Hôpitaux de Paris, Paris, France; 2 Université de Paris Cité, Paris, France; 3 Service de Néphrologie pédiatrique, Centre de Référence des Maladies Rénales Héréditaires de l’Enfant et de l’Adulte, centre de référence du syndrome néphrotique idiopathique de l'enfant et de l'adulte, Hôpital Necker-Enfants malades, Assistance Publique-Hôpitaux de Paris, Paris, France; 4 Service de Néphrologie pédiatrique, Hôpital Robert Debré, Assistance Publique-Hôpitaux de Paris, Paris, France; 5 Centre de Référence des Maladies Rares du Calcium et du Phosphore, Service de Néphrologie Rhumatologie Dermatologie Pédiatriques, Filières Santé Maladies Rares OSCAR, ORKID et ERKNet, Hôpital Femme Mère Enfant, Bron, France; 6 LYOS, Prévention des Maladies Osseuses, INSERM U1033, Université de Lyon, Lyon, France; 7 Faculté de Médecine Lyon Est, Université de Lyon, Lyon, France; 8 Service de Maladies Chroniques de l'enfant, Hôpital femme-enfants-adolescent, CHU Nantes, Nantes, France; 9 Service de Néphrologie Pédiatrique, Hopital Trousseau, APHP.6, DMU Origyne, Paris, France; 10 Service de pédiatrie multidisciplinaire, Assistance Publique-Hôpitaux de Marseille, Hopital de la Timone-Enfants, Marseille, France; 11 Service de néphrologie pédiatrique, Hôpital Jeanne-de-Flandre, CHRU de Lille, Lille, France; 12 Unité de Néphrologie Pédiatrique, Hôpital l’ Archet 2, CHU de Nice -, Nice, France; 13 Service de Néphrologie Pédiatrique, CHU de Montpellier, Montpellier, France; 14 Service de médecine infantile, secteur de néphrologie pédiatrique, hôpital d'Enfants de Brabois, CHRU de Nancy, Vandœuvre-lès-Nancy, France; 15 Service de néphropédiatrie, CHU de Tours, Hôpital Clocheville, Tours, France; 16 Department of Pediatric Nephrology, SoRare Reference Center, Toulouse University Hospital, Toulouse, France; 17 Service de pédiatrie, unité de néphrologie pédiatrique, centre de références des maladies rénales rares, CHU de Bordeaux, Bordeaux, France; 18 Service de pédiatrie, Centre hospitalier universitaire, Rouen, France; 19 Service de Chirurgie Pédiatrique, Hopital Universitaire de Caen, Caen, France; 20 Service d’Endocrinologie Pédiatrique, Hôpital Robert Debré, Assistance Publique-Hôpitaux de Paris, Paris, France; 21 Service de Médecine Génomique des Maladies Rares, Centre de Référence des Maladies Rénales Héréditaires de l’Enfant et de l’Adulte, Hôpital Universitaire Necker-Enfants malades, Assistance Publique-Hôpitaux de Paris, Paris, France; 22 Data Science Platform, INSERM UMR 1163, Imagine Institute, Université de Paris, Paris, France; 23 Centre de Recherche des Cordeliers, Sorbonne Université, INSERM, Université de Paris, Paris, France

**Keywords:** Denys-drash syndrome, Nephrotic syndrome, *WT1*

## Abstract

**Introduction:**

Denys-Drash syndrome (DDS) is a rare disease typically associated with a triad of early onset nephrotic syndromes (NS), susceptibility to Wilms tumor (WT), and genitourinary structural defects. DDS is caused by Wilms’ tumor suppression gene (*WT1*) variants, with the most frequent variants in exons 8 and 9. This study aimed to evaluate the long-term clinical outcomes and genotype-to-phenotype correlations in a large, multicenter cohort of children with typical DDS.

**Methods:**

We conducted a national retrospective study of all children diagnosed with a pathogenic variant in *WT1* exons 8 or 9 in France between 2000 and 2022.

**Results:**

Fifty-eight children with DDS and variants in exons 8 (*n* = 23) and 9 (*n* = 35) of the *WT1* gene were identified. Half of the children presented with NS (57% congenital, median age at presentation 0.3 years [interquartile range, IQR: 0.0–0.6]). Twenty-nine percent of children developed WT at a median age of 1.2 (0.5–2.2) years. Children with a variant in exon 8 developed NS much earlier than those with a variant in exon 9 (*P* = 0.0048), regardless of the type of genetic variation, leading to earlyier kidney failure (KF) (0.3 vs.1.4 years respectively; *P* = 0.0001) and higher mortality (35% vs 9%, *P* = 0.02). More than 90% of the truncating variants were located in exon 9 and were significantly associated with the occurrence of WT compared with the DNA-binding-site variants (*P* < 0.0015).

**Conclusion:**

In our cohort, children’s DDS clinical trajectory was associated with exon localization. In the era of genomic newborn screening, depicting genetic risk is of utmost importance for personalized patient care.

## Graphical abstract

**Figure:**
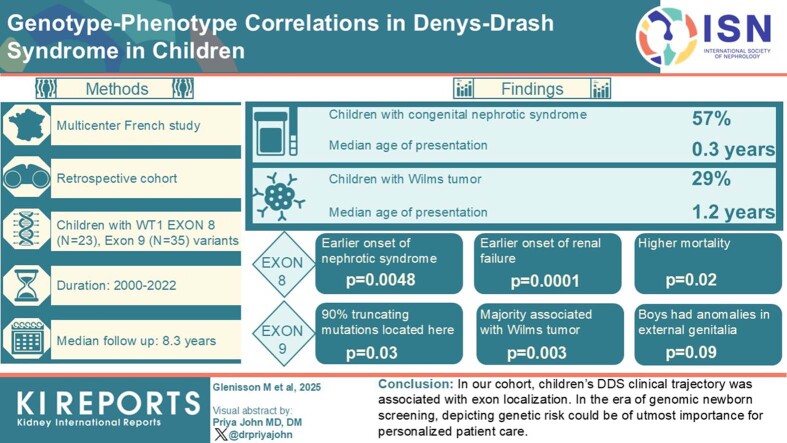

*WT1* is a tumor suppressor gene encoding the transcription factor Wilms’ Tumor 1, located in the 11p15 region, which plays a key role in kidney and gonadal development and is involved in tumorigenesis. The human *WT1* protein harbors a specific architecture containing an N-terminal region involved in transcription regulation and protein dimerization^1^^,^^2^ and a C-terminal domain comprising 4 tandemly repeated zinc fingers (ZF1–4) involved in interactions with DNA^3^^,^^4^ and RNA.^5^ Studies report that each ZF could play a specific role in DNA sequence discrimination and target binding (ZF2–4) or DNA binding affinity (ZF1).^3^^,^^6^^,^^7^

Anomalies in the *WT1* gene encompass a wide range of diseases, including Beckwith-Wiedemann syndrome, linked to epigenetic anomalies of the 11p15 region; WAGR syndrome (WT, aniridia, genitourinary anomalies, and mental retardation), linked to the deletion of the 11p region; and DDS and Frasier syndrome, linked to pathogenic variants of the *WT1* gene. These syndromes are characterized by a predisposition to WT and varying degrees of disorders of sex development (DSD).

Depending on the exon involved, pathogenic variants of the *WT1* gene can lead to DDS (Online Mendelian Inheritance in Man database identifier; OMIM#194080) or Frasier syndrome. Both conditions are characterized by progressive glomerulopathy and a predisposition to WT or DSD in 46 XY genotypes.^8^, ^9^, ^10^, ^11^, ^12^, ^13^, ^14^ *WT1* pathogenic variants causing DDS most often cluster in exons 8 and 9, which encode ZF2 and ZF3, respectively. Missense variants in these exons will either alter the ZF structure formed by 2 cysteines and 2 histidines (Cys2-His2 structure) or the sequence recognition amino acids at the protein-DNA interface (DNA-binding site).^15^ It has been suggested that the classical DDS triad could be partially explained by a genotype-to-phenotype correlation^16^; however, data from large cohorts are lacking. Correlation studies have indicated that truncating variants are associated with a higher risk of *WT*,^16^^,^^17^ whereas missense variants are more likely to cause NS. However, it is unclear how variants affect the Cys2-His2 structural amino acids compared with those affecting DNA-binding sites regarding their roles. Furthermore, it is unknown whether the localization of the variant in exons 8 or 9 influences the course of the disease.

In this study, we analyzed data from a large national cohort of children with DDS because of *WT1* pathogenic variants in exons 8 or 9 and aimed to evaluate whether genetic and/or clinical factors influence prognosis.

## Methods

### Patients

The study included children diagnosed with DDS who carried pathogenic variants in exons 8 or 9 (NM_024426.6) between 2000 and 2022 across 10 pediatric nephrology centers in France. The location variants in patients with DDS are presented in Supplementary Table S1).

To ensure the inclusion of all children in France with a molecular diagnosis of a pathogenic *WT1* variant and a clinical phenotype suggestive of a complete (clinical triad) or incomplete DDS during the study period, the selection process involved cross-referencing databases from genetics departments responsible for WT1 molecular analysis with records from pediatric nephrology departments that clinically managed these patients at a national level. We retrospectively collected clinical data using Dr. Warehouse,^18^ a comprehensive database containing 700,000 patients and 6 million health and medical reports. The database allows to search patients through structured data (biology) and free text (hospital reports). Congenital NS was defined as NS occurring before the age of 3 months.^19^ The study protocol was approved by the Hospital Necker-Enfants Malades Ethics Committee and complied with the Règlement Général sur la Protection des Données (French term for General Data Protection Regulation) regulatory rules. The terms “girl” and “boy” refer to phenotypic sex. For kidney transplantation (KTx), each center used its local immunosuppression schedule.

### Genetic Diagnosis

Molecular diagnosis was performed using Sanger sequencing of the *WT1* gene’s surrounding regions or high-throughput sequencing of a gene panel that includes *WT1* using the Twist Custom Panel Capture^20^ and Illumina sequence technology.

### Statistical Analyses

All analyses were performed using GraphPad Prism 9.4.1 software (GraphPad Software, San Diego). Continuous variables were expressed as median (IQR), and categorical variables were expressed as numbers (percentages). Variables were compared across groups using *t* test for normally distributed data and the Mann-Whitney U test for non-normally distributed data. Categorical variables were compared using Fisher's exact test. Survival without WT and NS was analyzed using Kaplan-Meier survival curves and log-rank tests. Missing data were excluded from analysis. When multiple comparisons were performed in the Kaplan-Meier analyses, the Bonferroni correction was applied with k = 6; therefore, *P* < 0.00833 was considered statistically significant.

## Results

During the study period, 58 children (43% males) with *WT1* variants in exon 8 (*n* = 23) and exon 9 (*n* = 35) were identified (Table 1). Most children (*n* = 26) had missense variants affecting a DNA-binding site. In addition, 9 other children had a missense variant affecting the Cys2-His2 structure of the ZFs. Twelve children had variants that did not affect the ZF structure. Among these, 3 children had variants that implicated a consensus splicing site with unknown functional effects. Eleven children presented with truncating variants, 91% of which were located in exon 9 (*P* = 0.03) (Table 1). All children had routine karyotypes; of the 33 children studied, 4 phenotypically female children had a 46 XY karyotype.

**Table 1:** Clinical features of patients with DDS related to exon 8 variants compared with exon 9 variants

Parameters	Total	Exon 8	Exon 9	*P*
*N*	58	23	35	-
Girl, *n* (%)	33 (57%)	15 (65%)	18 (51%)	0.41
Age at first clinical manifestation (yrs)	0.6 (0.04–1.1)	0.2 (0.01–0.6)	0.9 (0.3–1.6)	0.02
WT, *n* (%)	17 (29%)	3 (13%)	(14) 40%	0.003
Age at WT (yrs)	1.2 (0.5–2.2)	1.3 (1.0–2.2)	1.34 (0.8–2.6)	0.78
Anomalies of the external genitalia in boys, *n* (%)	23 (92%)	6 (75%)	17 (100%)	0.09
KF, *n* (%)	52 (90%)	22 (95%)	30 (86%)	0.39
Age at KF (yrs)	1.0 (0.3–1.7)	0.3 (0.1–0.9)	1.4 (0.6–2.3)	0.0001
Prophylactic nephrectomy, *n* (%)	28 (48%)	14 (60%)	14 (40%)	0.17
Age at transplantation (yrs)	3.6 (2.8–5.1)	3.2 (2.8–4.0)	4.2 (2.6–6.2)	0.25
Transplantation delay from KF (yrs)	2.2 (1.7–3.2)	2.1 (1.6–3.2)	2.4 (1.6–3.4)	1
Death, *n* (%)	11 (19%)	8 (35%)	3 (9%)	0.02
Type of variant				
Truncating, *n* (%)	11 (19%)	1 (4%)	10 (29%)	0.03
C2H2, *n* (%)	9 (16%)	5 (22%)	4 (11%)	0.70
DNA binding site, *n* (%)	26 (45%)	11 (48%)	15 (43%)	0.79
Others, *n* (%)	12 (21%)	6 (26%)	6 (17%)	0.33

C2H2, Cys2-His2 variant; DDS, Denys-Drash syndrome; KF, kidney failure; WT, Wilms’ tumor.

Continuous variables are expressed as median (interquartile range).

Categorical variables are expressed as numbers (percentages).

**Figure 1:**
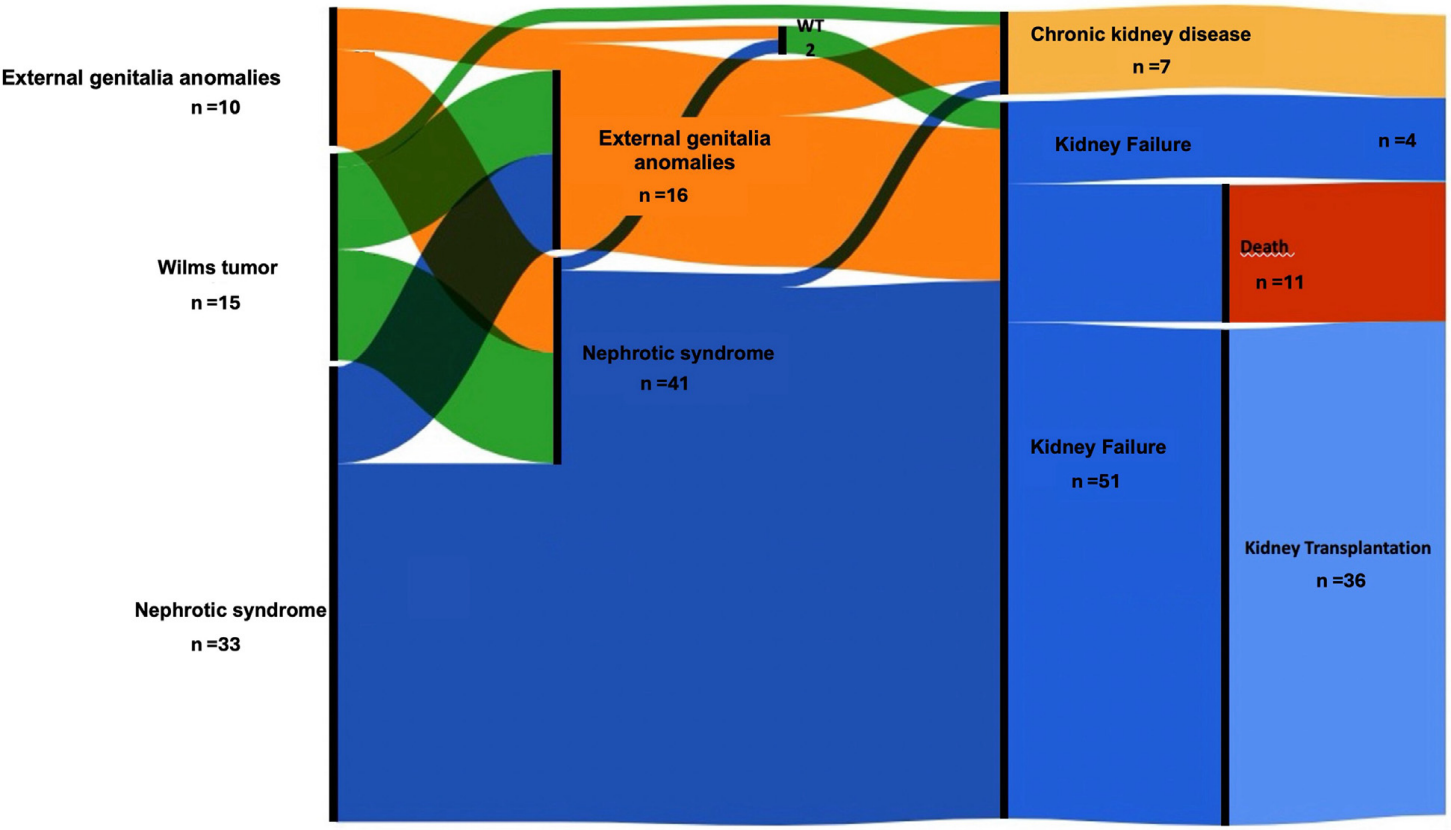
Alluvial diagram presenting the evolution of children with DDS. Children are depicted from their initial presentation (left) to their status at the last follow-up (right). DDS, Denys-Drash syndrome; WT, Wilms’ tumor.

**Figure 2:**
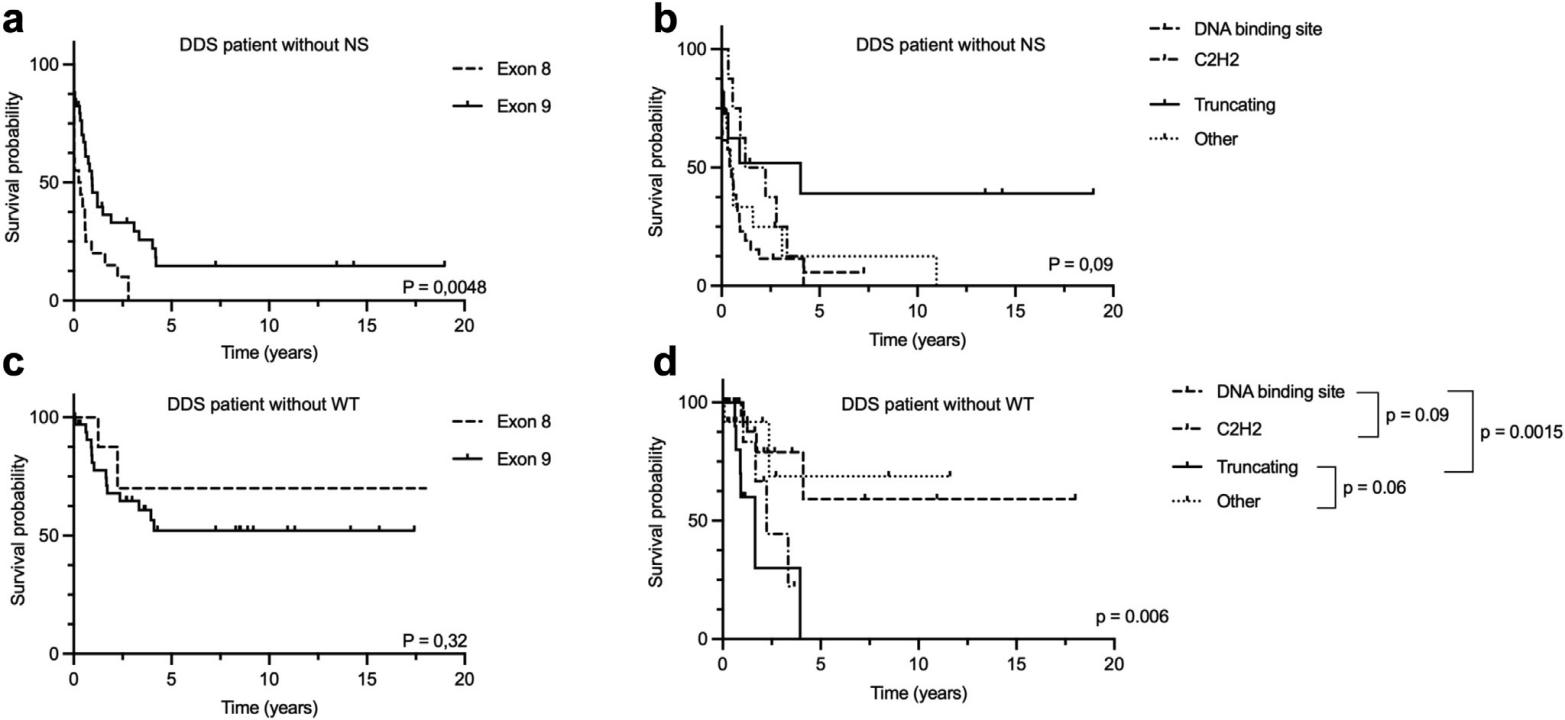
Kaplan-Meier kidney survival estimates in patients with DDS based on exon or *WT1* variant localization. (a) Without NS based on exon localization. (b) Without NS based on type of variant. (c) Non-*WT1* variants based on exon localization. (d) Non-*WT1* variants based on type of variant. *P* depending on log rank test. *C2H2*, Cis2-His2 variant; NS, nephrotic syndrome; WT, Wilms’ tumor.

Thirty-three children (73% girls) presented with NS at a median age of 0.3 (0–0.6) years (Figure 1), including 19 (57%) with congenital NS. Kidney biopsies at diagnosis were performed in 38 children, revealing diffuse mesangial sclerosis (DMS) as the most common histological finding in 16 children (42%), followed by focal segmental glomerulosclerosis in 7 (18%), tubulointerstitial lesions in 2, terminal kidney in 2, and nephrogenic rest in 1. Notably, children with a missense variant in exon 8 developed NS significantly earlier than those with a variant in exon 9 (*P* = 0.005, Figure 2a), regardless of the affected domain (*P* = 0.09, Figure 2b).

Seventeen children (29%) developed the WT (Supplementary Table S2). In 15 patients (88%), WT was the first clinical manifestation leading to a DDS diagnosis. The remaining 2 children had been previously diagnosed with DDS (1 with NS and the other with DSD). All patients with WT were diagnosed before the age of 4 years, with a median age of 1.2 (0.5–2.2) years. The sex ratio of the patients was balanced. Twelve children (70%) had proteinuria at the time of WT diagnosis, with 7 (41%) reaching the nephrotic range. WT was detected during ultrasonography monitoring in 2 children, whereas 14 (93%) presented with an abdominal mass. One child was diagnosed incidentally using ultrasonography for a urinary tract infection following urethroplasty for proximal hypospadias. Seven children (41%) had unilateral tumors, and 9 (53%) presented with synchronous bilateral disease at diagnosis (6 with bilateral WT, 3 with WT on one side, and suggestive lesions of nephroblastomatosis on the contralateral kidney). One child developed a metachronous WT. No child had metastasis at diagnosis. Children were treated according to the SIOP-2001 protocol^21^ until 2016, with more recent cases following the Umbrella protocol.^22^

Regarding the genotype-to-phenotype correlation, in the group of patients who developed WT, 15 of 17 had a variant in exon 9 (*P* = 0.03), including 6 patients with a truncating variant. There was no significant difference between the truncating and missense variants (*P* = 0.06). When comparing truncating variants more deeply with missense variant subgroups (DNA-binding site and Cys2-His2), patients with a variant in the DNA-binding site were less frequent among those who developed WT disease (*P* < 0.0015). At the same time, there was no difference with patients carrying variants in the Cys2-His2 domain (Figure 2d).

Twenty-three of the 25 boys (92%) in the cohort had anomalies of the external genitalia, including 8 who developed WT. The 2 boys without DSD had variants in exon 8 and did not develop WT but had KF. However, the localization of the variant within the exon did not appear to significantly affect the presence of external genital anomalies (*P* = 0.09; Table 1). External genitalia anomalies were diagnosed as the first clinical manifestation in 10 children (17%), with a median age of 0.7 (0.3–1.0) years. All boys who developed the WT (*n* = 8) presented with external genitalia abnormalities. In addition, 2 gonadoblastomas have been reported, and dysplasia was found on a testicular biopsy in 1 child with a variant in exon 8.

Median follow-up was 8.3 (5.2–14.6) years. Overall, 90% of children developed KF at a median age of 0.8 (0.3–1.6) years. Children without WT reached KF at a significantly younger age compared with those who developed WT (0.6 [0.2–1.5] years vs. 2.1 [1.3–5.8] years; *P* = 0.001) and had significantly lower survival rates (73% vs. 100%; Log-rank: *P* = 0.02) (Figure 2c and d). Furthermore, children with variants in exon 8 developed KF earlier than those with variants in exon 9 (0.3 vs.1.4 years, respectively, *P* = 0.0001) (Table 1). Notably, no correlation was observed between the type or localization of the variant and the timing of KF.

In total, 42 children (72%) underwent binephrectomy. Prophylactic nephrectomies were performed in 28 children with KF (48%) at a median age of 1.06 (IQR: 0.8–2.0) years. Children with WT had their last nephrectomy at a median age of 1.6 (IQR: 1.0–2.7) years. Histological examination revealed DMS in 23 children (55%), focal segmental glomerulosclerosis in 4 children (10%), nephrogenic rest in 10 children (23%), and missing data in 5 patients. Although children with a variant in exon 8 developed NS earlier than those with a variant in exon 9, the proportion of prophylactic binephrectomies did not differ between the groups (37% in the exon 9 group and 56% in the exon 8 group; *P* = 0.17).

Only 1 child with WT received a preemptive kidney transplant from a living donor before undergoing nephrectomy. For this child, 1 nephrectomy was performed simultaneously with the KTx and the subsequent nephrectomy was performed 1 month later. Among the 5 children with truncating variants who did not develop WT in our cohort, 3 underwent bilateral nephrectomy before the age of 1.2 years, 1 died at the age of 3 years before undergoing contralateral nephrectomy, and only 1 did not undergo nephrectomy.

In our cohort, 36 children underwent KTx at a median age of 3.8 (IQR: 2.8–5.1) years. Twenty-seven children (75%) received kidneys from deceased donors. At the end of the follow-up period, 2 children remained on the waiting list for KTx. Median delay from KF to KTx was 2.2 (IQR: 1.7–3.2) years. The time between diagnosis and KT was significantly longer in children with WT (3.3 vs. 2.4 years, *P* = 0.01), although the delay from KF to KTx was similar between the 2 groups (2.8 vs. 2.0 years, *P* = 0.19). During the follow-up period, we did not observe any changes in the delay between nephrectomy and transplantation.

At the end of the follow-up period, 7 children still had their native kidneys. One child with KF undergoing hemodialysis retained his native kidneys because of the parental refusal of nephrectomy despite severe hypertension. He did not develop WT at the latest follow-up at the age of 9 years. Three children with DSD were treated for non-nephrotic proteinuria, whereas 3 children with WT did not experience KF. Three children experienced KF without their native kidneys and are awaiting transplantation. One child did not continue with the follow-up process.

Eleven children (19%) died during the follow-up period at a median age of 0.3 years (IQR: 0.1–0.7). The mortality rate was higher in children with exon 8 variants (35% vs. 9%, *P* = 0.02) than in those with exon 9 variants. There were no significant differences in mortality rates over time.

## Discussion

To the best of our knowledge, we present the largest cohort of children with DDS to date, providing detailed clinical characteristics to evaluate genotype-to-phenotype correlations. We exclusively focused on children with variants in exons 8 and 9 to obtain a more homogeneous population of children with DDS. Our findings suggest that the variant’s localization, whether in exon 8 or 9, may influence the phenotype and outcomes and should be considered when evaluating a child.

Children with a pathogenic variant in exon 8 exhibit earlier disease onset, primarily manifesting as NS, in contrast to those with a variant in exon 9. This finding aligns with that of a recent study of 75 Chinese children;^23^ however, it differs in that our study did not include children with intron 9 variants, leading to the loss of the *WT1* isoform associated with Frasier syndrome, which has a later onset and slower progression than DDS. Nagano *et al.*^17^ reported that children with variants affecting the DNA-binding site had a more severe phenotype than those with variants affecting the Cys2-His2 structural amino acids. Reanalysis of Nagano *et al.*’s study, which included 161 pediatric patients (Supplementary Figure S1), supports our finding that children with missense variants in exon 8 develop symptoms earlier than those with variants in exon 9 (*P* = 0.02).^17^ Altogether, these data strongly suggest that variant localization and the affected ZF domains affect the disease course. It has been described that the 4 ZFs of *WT1* may have a different impact on DNA binding. The 4 ZFs of *WT1* may differently affect DNA binding, with ZF1 reportedly having a regulatory effect.^24^ It is plausible that ZF2 and ZF3 also play distinct roles, which could explain the influence of variant localization on disease progression.

The structure of the *WT1* gene is complex, and studies have correlated the type of variant types (truncating, missense affecting DNA-binding site or Cys2-His2 structure, and others) with disease trajectory, particularly the occurrence of WT with truncating variants.^16^ This association is well-documented in children with isolated WT and truncating variants in the *WT1* gene.^25^ Our study reports similar variant proportions as previous studies (Supplementary Table S1).^16^^,^^17^^,^^23^ Specifically, 54% of children with truncating variants (6/11, 55%) developed WT, a rate similar to those with Cys2-His2 domain variants (5/9 children, 55%) but significantly higher than those with DNA-binding site variants (4/26 children, 15%; *P* = 0.0015). Notably, more cases were observed in the exon 9 group, which was associated with the enrichment of truncating variants (29%) compared with exon 8 (4%). Thus, it was difficult to determine whether the occurrence of WT in our cohort was primarily linked to exon 9 or truncating variants alone, as suggested by previous studies. The high mortality rate in children with exon 8 variants complicates the evaluation of tumor risk. Similarly, prophylactic nephrectomy may be a confounding factor, although the difference between the 2 groups was not significant.

In our study, all children had proteinuria, ranging from nonnephrotic proteinuria to NS, contrasting with Lehnhardt *et al.*, who reported proteinuria in 88% of the children with DDS.^26^ Regarding histological lesions, 42% of kidney biopsies in our study revealed DMS, consistent with Lehnhardt *et al.*’s series.^26^ Our study showed a higher rate of DMS than focal segmental glomerulosclerosis lesions as in Roca *et al.*’s cohort.^27^ This difference may be explained by 82% of our children having missense variants. Similarly, DMS was predominant in children with missense variants in a study by Lipska *et al.*^16^ As indicated in recent guidelines, genetic analyses are indicated as a first-line investigation in the case of congeniral nephrotic syndrome or syndromic nephrotic syndrome, such as DDS, without performing a kidney biopsy.^19^^,^^28^ If genetic analyses are unavailable or do not yield any causative variants, a kidney biopsy may be indicated. Thirty-eight patients underwent renal biopsy; among them, 13 had congenital NS, which was high. However, this can be explained by the era of diagnosis for these children because genetic test had longer results delay and by the severity of these children because 7 of them died.

Only 2 children who developed WT had a known *WT1* pathogenic variant before diagnosis and benefited from WT screening. For the others, the diagnosis was based on a palpable mass in 14 of 15 cases, and nephrotic-range proteinuria was already present. Of note, most children with NS who never developed WT died of KF at an age preceding the median age of WT presentation (median age at death: 0.3 [IQR: 0.1–0.7] years). No WT cases were diagnosed after the onset of KF. This observation may be attributed to the fact that children with KF underwent prophylactic nephrectomies as recommended^19^ in 28 of 58 cases, at a median age younger than the median age of WT diagnosis (1.06 vs. 1.20 years). Only 1 child with KF retained native kidneys beyond the age of 4 years, which is the maximum observed age for WT presentation, after their parents declined prophylactic nephrectomy. At the last follow-up, the child had not developed WT at the age of 9 years.

Nephrectomy, whether performed at the stage of KF or for oncological reasons, interferes with the natural history of the disease, complicating the assessment of tumor risk and the rate of progression to KF. There are no clear recommendations on the optimal age for nephrectomy in DDS when NS is present. Unilateral or bilateral nephrectomy may be considered in children with severe complications, such as failure to thrive, thrombosis, or difficulty in maintaining intravascular euvolemia,^19^ and before KTx.^19^ In our cohort, children without WT underwent bilateral nephrectomy before or at about the age of 1 year , which is earlier than that reported by Lipksa *et al.*,^16^ in which all children with exonic variants had bilateral nephrectomies before the age of 5 years. Before prophylactic nephrectomy, WT screening using ultrasound every 3 months is recommended for children with exonic variants until the age of 7 years.^29^

Our study has several limitations. Because this was a retrospective study, it was inherently constrained by incomplete data in some cases. Patient inclusion was based on cross-referencing records from the genetics and French pediatric nephrology departments, leading to loss of follow-up. Despite the broad scope of our selection process, a selection bias remains a possibility. Specifically, we may have missed ultrarare cases of isolated WT managed exclusively in pediatric oncology centers without the involvement of nephrology centers. However, as highlighted by Van Peer *et al.*,^30^ such cases are typically associated with WT1 variants located outside the hotspot region targeted in this study and thus fall beyond the scope of this study.

We recruited children from specialized pediatric nephrology centers, which may introduce selection bias, particularly because some centers focus on congenital NS.

With the increasing use of genetic testing as an initial approach for detecting external genital abnormalities, boys with *WT1* variants are likely to be diagnosed before the development of WT. This subgroup may benefit from screening for proteinuria and WT. Early detection of proteinuria and the introduction of nephroprotective medications could lead to a better kidney prognosis. The rationale for WT surveillance relies on early diagnosis at a smaller tumor size, enabling nephron-sparing surgery and lower-stage disease at diagnosis, which improves survival and reduces treatment-related toxicity.^29^^,^^31^ Unfortunately, girls remain the principal subgroup without access to early diagnosis unless they develop NS. In the era of genomic newborn screening, the early identification of actionable tumor genes is essential, because it allows for timely intervention, making tumors more treatable and enabling tailored patient management to improve the prognosis and quality of care.

Our study underscores the critical role of phenotypic and molecular characterization in children with DDS, demonstrating that variants located in exons 8 or 9 may influence disease progression. A detailed phenotypic assessment facilitates early molecular diagnosis and enables precise oncological monitoring. If our results are validated by other studies, they could have significant implications in clinical practice, particularly in the oncological management of patients with exon 9 variants.

## Disclosure

All the authors declared no competing interests.

## Supplementary Material

Supplementary File (PDF) **Figure S1.** Comparison of the age at diagnosis between children with variants in exon 8 and exon 9 in the Nagano’s cohort. **Table S1.** Location variants in patients with Denys-Drash syndrome. **Table S2.** Clinical features of patients with Denys-Drash syndrome related to their Wilm’s tumor status. STROBE Checklist.
