# Corrigendum to “Genotype-Phenotype Correlations in Denys-Drash Syndrome in Children” [*Kidney International Reports* Volume 10, Issue 4, April 2025, Pages 1205-1212]

**DOI:** 10.1016/j.ekir.2025.05.001

**Published:** 2025-05-04

**Authors:** Mathilde Glénisson, Mathilde Grapin, Thomas Blanc, Evgenia Preka, Julien Hogan, Manon Aurelle, Gwenaëlle Roussey, Antoine Mouche, Caroline Rousset-Rouviere, Robert Novo, Camille Faudeux, Marc Fila, Isabelle Vrillon, Sylvie Cloarec, Thomas Simon, Jérôme Harambat, Edouard Martinez Casado, Julien Rod, Morgane Carre Lecoindre, Laetitia Martinerie, Laurence Heidet, Olivia Boyer, Nicolas Garcelon, Jessica Kachmar, Guillaume Dorval, Sabine Sarnacki

**Affiliations:** 1 Service de chirurgie viscérale, urologie et transplantation, Hôpital Necker-Enfants malades, GH Centre, Assistance Publique-Hôpitaux de Paris, Paris, France; 2 Université de Paris Cité, Paris, France; 3 Service de Néphrologie pédiatrique, Centre de Référence des Maladies Rénales Héréditaires de l’Enfant et de l’Adulte, centre de référence du syndrome néphrotique idiopathique de l’enfant et de l’adulte, Hôpital Necker-Enfants malades, Assistance Publique-Hôpitaux de Paris, Paris, France; 4 Service de Néphrologie pédiatrique, Hôpital Robert Debré, Assistance Publique-Hôpitaux de Paris, Paris, France; 5 Centre de Référence des Maladies Rares du Calcium et du Phosphore, Service de Néphrologie Rhumatologie Dermatologie Pédiatriques, Filières Santé Maladies Rares OSCAR, ORKID et ERKNet, Hôpital Femme Mère Enfant, Bron, France; 6 Service de Maladies Chroniques de l’enfant, Hôpital femme-enfants-adolescent, CHU Nantes, Nantes, France; 7 Service de Néphrologie Pédiatrique, Hopital Trousseau, APHP.6, DMU Origyne, Paris, France; 8 Service de pédiatrie multidisciplinaire, Assistance Publique-Hôpitaux de Marseille, Hopital de la Timone-Enfants, Marseille, France; 9 Service de néphrologie pédiatrique, Hôpital Jeanne-de-Flandre, CHRU de Lille, Lille, France; 10 Unité de Néphrologie Pédiatrique, Hôpital l’ Archet 2, CHU de Nice -, Nice, France; 11 Service de Néphrologie Pédiatrique, CHU de Montpellier, Montpellier, France; 12 Service de médecine infantile, secteur de néphrologie pédiatrique, hôpital d’Enfants de Brabois, CHRU de Nancy, Vandœuvre-lès-Nancy, France; 13 Service de néphropédiatrie, CHU de Tours, Hôpital Clocheville, Tours, France; 14 Department of Pediatric Nephrology, SoRare Reference Center, Toulouse University Hospital, Toulouse, France; 15 Service de pédiatrie, unité de néphrologie pédiatrique, centre de références des maladies rénales rares, CHU de Bordeaux, Bordeaux, France; 16 Service de pédiatrie, Centre hospitalier universitaire, Rouen, France; 17 Service de Chirurgie Pédiatrique, Hopital Universitaire de Caen, Caen, France; 18 Service d’Endocrinologie Pédiatrique, Hôpital Robert Debré, Assistance Publique-Hôpitaux de Paris, Paris, France; 19 Service de Médecine Génomique des Maladies Rares, Centre de Référence des Maladies Rénales Héréditaires de l’Enfant et de l’Adulte, Hôpital Universitaire Necker-Enfants malades, Assistance Publique-Hôpitaux de Paris, Paris, France; 20 Data Science Platform, INSERM UMR 1163, Imagine Institute, Université de Paris, Paris, France; 21 Centre de Recherche des Cordeliers, Sorbonne Université, INSERM, Université de Paris, Paris, France

The authors regret that co-author Laetitia Martinerie was missing from the published manuscript.

The authors apologize for any inconvenience caused.

DOI of original article: https://doi.org/10.1016/j.ekir.2025.01.014

