# Supplementary material for: Genotype-Phenotype Correlations in Denys-Drash Syndrome in Children
Source: Kidney Int Rep. 2025 Jan 16;10(4):1205–12. doi: 10.1016/j.ekir.2025.01.014 (PMC12034853; doi:10.1016/j.ekir.2025.01.014)
Supplement: Supplementary File (PDF) — Figure S1. Comparison of the age at diagnosis between children with variants in exon 8 and exon 9 in the Nagano’s cohort. Table S1. Location variants in patients with Denys-Drash syndrome. Table S2. Clinical features of patients with Denys-Drash syndrome related to their Wilm’s tumor status. STROBE Checklist. [file mmc1.pdf]

Supplementary :

Table S1 : Location variants in DDS patients :

Patients	Variants	Location
1	c.1399C>T	DNA binding site
2	c.1405G>A	Other
3	c.1354+5G>A	Other
4	c.1406A>T	Other
5	c.1336C>T	C2H2
6	c.1323C>A	DNA binding site
7	c.1338C>A	C2H2
8	c.1400G>A	DNA binding site
9	c.1384C>T	Truncating
10	c.1400G>A	DNA binding site
11	c.1357+1G>A	Other
12	c.1315C>T	DNA binding site
13	c.1316G>A	DNA binding site
14	c.1316G>A	DNA binding site
15	c.1333A>G	DNA binding site
16	c.1316G>A	DNA binding site
17	c.1316G>A	DNA binding site
18	c.1405G>A	Other
19	c.1349A>G	C2H2
20	c.1372T>C	C2H2
21	c.1352C>T	Other
22	c.1400G>A	DNA binding site
23	c.1399C>T	DNA binding site
24	c.1399C>T	DNA binding site
25	c.1399C>T	DNA binding site
26	c.1315 C>T	DNA binding site
27	c.1399C>T	DNA binding site
28	c.1447+2T>A	Other
29	c.1301 G>A	Other
30	c.1408C>T	DNA binding site
31	c.1316G>A	DNA binding site
32	c.1315C>T	DNA binding site
33	c.1319G>A	Other
34	c.1384C>T	DNA binding site
35	c.1399C>T	DNA binding site
36	c.1405G>A	Other
37	c1288C>T	Truncating
38	c.1399C>T	DNA binding site

39	c.1387C>T	Truncating
40	c.1411delC	Truncating
41	c.1325A>C	Other
42	c.1387 C>T	Truncating
43	c.1373G>A	C2H2
44	c.1421A>G	C2H2
45	c.1431del	Truncating
46	c.1399C>T	DNA binding site
47	c.1387C>T	Truncating
48	c.1421A>G	C2H2
49	c.1406A>T	Other
50	c.1387C>T	Truncating
51	c. 1387 C>T	Truncating
52	c. 1387 C>T	Truncating
53	c.1399C>T	DNA binding site
54	c. 1387 C>T	Truncating
55	c.1349A>T	C2H2
56	c.1399C>T	DNA binding site
57	c.1405G>A	Other
58	c.1316G>A	DNA binding site

Table S2 : Clinical features of patients with DDS related to their Wilm's tumor status

	**Total**	**WT +**	**WT -**	**p**
**N**	58	17	41	-
**Girls (N,%)**	23 (39%)	9 (53%)	24 (58%)	0.77
**Anomalies of the external genitalia in boys** (N, %)	23 (92%)	8 (100%)	15 (88%)	1
**Age at first clinical manifestation** (years)	0.6 (0.04-1.1)	1.2 (0.5-2.7)	0.3 (0.01-0.9)	**0.005**
**Age at KF** (years)	1.0 (0.3-1.7)	2.1 (1.3-5.8)	0.6 (0.2-1.5)	**0.001**
**Age at kidney transplantation (**years)	3.6 (2.8-5.1)	4.7 (3.8-7.7)	2.9 (2.4-4.2)	**0.003**
**Transplantation delay from KF (years)**	2.2 (1.7-3.2)	2.8 (2.0-3.2)	02.0 (1.5-3.4)	0.19
**Death (N, %)**	11 (19%)	0 (0%)	11 (27%)	**0.02**
**Localization of variant**				
Exon 8/9 (N, %)	23 (40%) / 35 (60%)	2 (12%) /15 (88%)	21 (51%) / 20 (49%)	**0.007**
**Type of variant**				
**Truncating** (N, %)	11 (19%)	6 (35%)	5 (12%)	0.06
**C2H2** (N,%)	9 (15%)	5 (29%)	4 (10%)	0.13
**DNA Binding Site** (N,%)	26 (44%)	4 (23%)	22 (54%)	**0.04**
**Others** (N, %)	12 (21%)	2 (12%)	10 (24%)	0.48

C2H2: Cys2-His2 variant KF: Kidney Failure; WT: Wilms' Tumor
Values in years are expressed as median with interquartile range

Figure S1 : Comparison of the age at diagnosis between children with variants in exon 8 and exon 9 in the nagano's cohort.

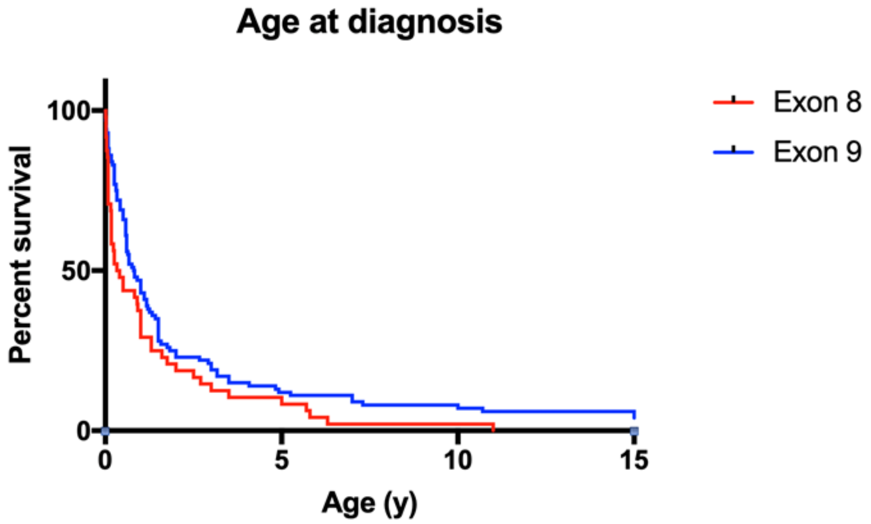

Gehan-Breslow-Wilcoxon test p=0.02

	ex8	ex9	p
Total	53	108	
C2H2	16	19	0,10
DBS	29	59	1
Autre	8	30	0,08

STROBE Statement—Checklist of items that should be included in reports of ***cohort studies***

	Item No	Recommendation	Page No
**Title and abstract**	1	(*a*) Indicate the study's design with a commonly used term in the title or the abstract<br>(*b*) Provide in the abstract an informative and balanced summary of what was done and what was found	1 - 3<br>3
**Introduction**			
Background/rationale	2	Explain the scientific background and rationale for the investigation being reported	4
Objectives	3	State specific objectives, including any prespecified hypotheses	4
**Methods**			
Study design	4	Present key elements of study design early in the paper	5
Setting	5	Describe the setting, locations, and relevant dates, including periods of recruitment, exposure, follow-up, and data collection	5
Participants	6	(*a*) Give the eligibility criteria, and the sources and methods of selection of participants. Describe methods of follow-up<br>(*b*) For matched studies, give matching criteria and number of exposed and unexposed	5
Variables	7	Clearly define all outcomes, exposures, predictors, potential confounders, and effect modifiers. Give diagnostic criteria, if applicable	5-6
Data sources/ measurement	8*	For each variable of interest, give sources of data and details of methods of assessment (measurement). Describe comparability of assessment methods if there is more than one group	5
Bias	9	Describe any efforts to address potential sources of bias	/
Study size	10	Explain how the study size was arrived at	/
Quantitative variables	11	Explain how quantitative variables were handled in the analyses. If applicable, describe which groupings were chosen and why	5-6
Statistical methods	12	(*a*) Describe all statistical methods, including those used to control for confounding<br>(*b*) Describe any methods used to examine subgroups and interactions<br>(*c*) Explain how missing data were addressed<br>(*d*) If applicable, explain how loss to follow-up was addressed<br>(*e*) Describe any sensitivity analyses	5-6<br>/<br>5-6<br>/
**Results**			
Participants	13*	(a) Report numbers of individuals at each stage of study—eg numbers potentially eligible, examined for eligibility, confirmed eligible, included in the study, completing follow-up, and analysed<br>(b) Give reasons for non-participation at each stage<br>(c) Consider use of a flow diagram	6
Descriptive data	14*	(a) Give characteristics of study participants (eg demographic, clinical, social) and information on exposures and potential confounders<br>(b) Indicate number of participants with missing data for each variable of interest<br>(c) Summarise follow-up time (eg, average and total amount)	6;16<br>6;7;8<br>8;9
Outcome data	15*	Report numbers of outcome events or summary measures over time	6;7;8;9

Main results	16	(*a*) Give unadjusted estimates and, if applicable, confounder-adjusted estimates and their precision (eg, 95% confidence interval). Make clear which confounders were adjusted for and why they were included<br>(*b*) Report category boundaries when continuous variables were categorized<br>(*c*) If relevant, consider translating estimates of relative risk into absolute risk for a meaningful time period	/<br>6;7;8;9<br>/
Other analyses	17	Report other analyses done—eg analyses of subgroups and interactions, and sensitivity analyses	6;7;8;9
**Discussion**			
Key results	18	Summarise key results with reference to study objectives	9;10;11;12
Limitations	19	Discuss limitations of the study, taking into account sources of potential bias or imprecision. Discuss both direction and magnitude of any potential bias	11
Interpretation	20	Give a cautious overall interpretation of results considering objectives, limitations, multiplicity of analyses, results from similar studies, and other relevant evidence	11;12
Generalisability	21	Discuss the generalisability (external validity) of the study results	12
**Other information**			
Funding	22	Give the source of funding and the role of the funders for the present study and, if applicable, for the original study on which the present article is based	13

*Give information separately for exposed and unexposed groups.

Note: An Explanation and Elaboration article discusses each checklist item and gives methodological background and published examples of transparent reporting. The STROBE checklist is best used in conjunction with this article (freely available on the Web sites of PLoS Medicine at http://www.plosmedicine.org/, Annals of Internal Medicine at http://www.annals.org/, and Epidemiology at http://www.epidem.com/). Information on the STROBE Initiative is available at http://www.strobe-statement.org.
